# Online real-time teaching interaction and self-regulated learning ability: a cross-sectional mediation study among undergraduate nursing students

**DOI:** 10.3389/fpubh.2026.1872098

**Published:** 2026-09-07

**Authors:** Dongmei Liu, Wanpeng Zhen, Meiyuan Yang, Yongchao Jin, Ye Lin, Huifeng Bo, Rui Yang

**Affiliations:** 1 School of Science, North China University of Science and Technology, Tangshan, China; 2 College of Civil and Architectural Engineering, North China University of Science and Technology, Tangshan, China; 3 Logistics Service Center, North China University of Science and Technology, Tangshan, China; 4 School of Philosophy and Sociology, Jilin University, Changchun, China

**Keywords:** academic self-efficacy, mediating role, nursing students, online real-time teaching interaction, self-regulated learning ability

## Abstract

**Background:**

As higher education increasingly shifts toward online models, nursing education has also widely adopted digital learning formats, one of the key theoretical foundations of which is the self-regulated learning model. Existing research has confirmed that academic self-efficacy is significantly and positively associated with the development of students’ self-regulated learning abilities. However, its mediating mechanism between online real-time teaching interaction and self-regulated learning ability in nursing students has not yet been systematically explored.

**Methods:**

This study adopted a cross-sectional survey design. From September 30th, 2025 (with a data collection period of one week), a questionnaire survey was conducted among nursing students from four nursing undergraduate programs in Jilin Province. Using the convenience sampling method, a total of 813 valid questionnaires were collected. Data were collected through questionnaires, utilizing the following instruments: the online real-time teaching interaction (ORTTI), the academic self-Efficacy (ASE), and the self-regulated learning (SRL) scales. All data were processed using the SPSS AU statistical analysis platform. Analytical methods encompassed descriptive statistics, correlation analysis between variables, path analysis with Bootstrap mediation testing, and mediation effect testing for academic self-efficacy.

**Results:**

The results indicate that, the online real-time teaching interaction was significantly and positively related to self-regulated learning ability in nursing students (*r* = 0.809, *p* < 0.01). Academic self-efficacy appears to partially mediate the relationship between online real-time teaching interaction and self-regulated learning ability. Its mediating effect value is 0.478, accounting for 56.7% of the total effect.

**Conclusion:**

These findings suggest that online real-time teaching interaction is directly and positively associated with the self-regulated learning ability of nursing students, and may also be indirectly associated with it through higher academic self-efficacy. The results highlight the importance of intentionally designing interactive components in online nursing education that support academic self-efficacy. These findings may inform the design of interactive online courses that support nursing wstudents’ academic self-efficacy and self-regulated learning.

## 1 Introduction

Online learning has gradually integrated into contemporary education and has become an essential component of it. Its advantages lie in providing flexibility, wide accessibility and personalized learning experiences (1). The self-regulated learning (SRL) model provides one of the important theoretical frameworks for online learning. This model emphasizes that metacognitive skills, motivation, and self-regulation strategies play a crucial role in achieving a good learning experience (2). SRL is defined as the active and constructive self-regulation ability demonstrated by learners in the process of setting goals, monitoring the learning process, and regulating their cognition, motivation and behavior (3). In an online learning environment, the core features of SRL include metacognitive skills (such as learning planning, process monitoring and evaluation), time management, environmental structuring, perseverance and the ability to seek help (4). Zimmerman noted that SRL is a dynamic and complex process with a structure, mainly divided into three core stages: preparatory thinking, behavioral execution, and self-reflection. During the pre-thinking stage, learners set learning goals and plan corresponding strategies (5). In the execution stage, learners implement these strategies, monitor learning progress, and employ self-regulatory strategies such as time management (6). Finally, in the self-reflection stage, learners evaluate their learning performance and identify reasons for success or failure, thereby providing feedback and guidance for future learning (7). These stages do not occur once but are cyclical, supporting learners’ continuous learning and lifelong learning (5).

The effectiveness of online learning largely depends on learners’ ability to plan and monitor their own learning behaviors. Whether students can achieve good learning outcomes in the online education environment mainly depends on the development level of their SRL ability (8). Strong SRL abilities are not only associated with higher learning engagement (9) but also correlate positively with online learning efficiency and academic achievement (10). The development of SRL abilities is associated with multiple educational environmental factors, including teaching methods, learning resources, assessment practices (11), students’ perceptions of teacher support (12), and peer learning atmospheres (13). An educational environment that encourages active teacher-student interaction, collaborative learning, and mutual respect facilitates the development of students’ SRL abilities. In this process, expectations conveyed by teachers through instructional interactions can be internalized by students as sustained learning motivation (14). Correspondingly, students with a high level of self-regulation ability also exhibit a more acute perception and adaptability to the physical and social learning environment.

Although online education has shown great potential with its flexibility and accessibility, its effectiveness largely depends on the quality of course teaching design and teaching interaction. The lack of teaching interaction can easily lead to a decline in students’ learning motivation, insufficient participation and weakened collaboration ability (15). In addition, technical obstacles such as unstable Internet connection, limited devices and uneven digital resources can also reduce students’ learning experience (16, 17). More importantly, the online learning environment requires students to have a high degree of self-regulation ability. In the absence of structured guidance and support, learners are prone to cognitive overload, ambiguous goals and difficulties in self-regulation (15). Therefore, online teaching should incorporate real-time interaction mechanisms to effectively support students’ SRL in the online learning environment (18). According to Schunk (3), the effectiveness of online learning largely depends on learners’ ability to plan and monitor their own learning behaviors. Whether students can achieve good learning outcomes in the online education environment mainly depends on the development level of their SRL ability (8). Strong SRL abilities not only associated with higher learning engagement (9). Online real-time teaching interaction includes various forms such as real-time discussions, immediate feedback, and collaborative problem-solving. According to Schunk, such interactions can effectively enhance students’ academic self-efficacy by providing alternative experiences and verbal encouragement from the teacher (3).

Self-efficacy is a core concept in social cognitive theory. Bandura defined it as an individual’s judgment and belief regarding their ability to successfully complete a specific task (19). This belief profoundly contributes to an individual’s behavioral performance, shaping their confidence in task completion and coping strategies (20). Particularly in challenging situations like adapting to new learning environments, self-efficacy plays a decisive role in individual success (21). Multiple studies reveal a strong connection between self-efficacy and SRL. Self-efficacy and self-regulation capabilities jointly form key elements for achieving academic goals, underscoring the value of instructional strategies that support students’ related skills (22). Zheng and Xiao’s research has confirmed that online self-efficacy has a significant impact on the self-regulation levels in language learning, participation in informal digital learning, and course satisfaction (23). Agustiani et al. (24) similarly identified a significant positive correlation among self-efficacy, SRL, and academic achievement. Similarly, Dogu et al. observed a significant positive correlation between nursing students’ self-efficacy and self-regulation abilities in a clinical teaching environment (25).

In summary, online real-time teaching interaction is closely associated with students’ SRL level directly or indirectly through the mediating variable of academic self-efficacy. However, most existing studies have focused on ordinary online learners and non-health-related professional groups, and have not clarified the interaction relationship among online real-time teaching interaction, academic self-efficacy, and nursing students’ SRL. Conducting related research is an important prerequisite for building a scientific and complete online nursing education system and supporting students’ learning development and clinical ability optimization.

According to the triadic interaction theory of social cognition (26), there is a dynamic interrelationship of mutual influence among the environment (online real-time teaching interaction), the individual (academic self-efficacy), and the behavior (SRL). Teaching interaction, as an environmental variable, first enhances the learners’ self-efficacy through providing successful experiences and alternative experiences (19); self-efficacy then influences the learners’ goal setting, strategy use, and self-monitoring and other self-regulatory behaviors (5). Therefore, academic self-efficacy may play a mediating role between online real-time teaching interaction and SRL. Based on this, this study proposes the following hypotheses:

Hypothesis 1: Online real-time teaching interaction is significantly and positively associated with self-regulated learning ability in nursing students.

Hypothesis 2: Academic self-efficacy is positively associated with self-regulated learning ability in nursing students.

Hypothesis 3: Online real-time teaching interaction is significantly and positively associated with academic self-efficacy in nursing students.

Hypothesis 4: Academic self-efficacy mediates the relationship between online real-time teaching interaction and self-regulated learning ability in nursing students.

## 2 Materials and methods

### 2.1 Participants

Participants were recruited from four nursing-program-offering higher education institutions in Jilin Province, with a total population of approximately 3,000 nursing students. Using the convenience sampling method, electronic questionnaires were distributed to the nursing students of the 4 institutions through the Wenguanxing platform. No random sampling was conducted, all students who voluntarily participated were included in the analysis. The inclusion criteria were as follows: nursing students currently enrolled in the program, participating in online course learning, and voluntarily participating in this study upon obtaining informed consent. The exclusion criteria were: students who are out of school or on leave of absence due to illness or other personal reasons. This research was approved by the Ethics Committee of North China University of Technology (2025090) on September 29th, 2025. All participants were required to read the electronic informed consent form before officially filling out the questionnaire. The informed consent form clearly stated the research purpose, the principle of voluntary participation, anonymity, and data confidentiality measures. Participants clicked the “Agree” button to enter the questionnaire filling page, which was regarded as the confirmation of digital informed consent. Those who did not click “Agree” could not continue to fill out the questionnaire. All respondents were aware and voluntarily agreed to participate in this study. The survey ran for one week from September 30th, 2025, yielding 850 responses. After quality control screening, 37 invalid questionnaires were eliminated (The answer is incomplete, the completion time is extremely short, and the same IP address or the same device submits or answers in a repetitive manner multiple times within a short period of time). A total of 813 valid questionnaires were retained for statistical analysis, yielding an effective response rate of 95.65%. The sample size of this study is 813, which is more than 10 times the number of observed variables. This fully meets the statistical requirements for structural equation modeling (SEM) and mediation effect analysis. In the questionnaire design, all the questions were set as “mandatory questions,” and there was no “skip” or “fill later” function. Therefore, during the original data collection process, it was impossible to submit the questionnaire with any missing items. As a result, the 813 valid questionnaires retained did not have any missing values.

### 2.2 Measures

#### 2.2.1 Online real-time teaching interaction scale (ORTTI)

The online real-time teaching interaction scale (ORTTI) adopts the version compiled by Cheng et al. (27). This scale was initially constructed through literature research methods. After expert review and pre-testing, four dimensions were finally formed, consisting of 32 items: human-computer interaction (5 items, score range 5 ~ 25), teacher-student interaction (9 items, score range 9 ~ 45), student–student interaction (9 items, score range 9 ~ 45), and self-interaction (9 items, score range 9 ~ 45). The scale adopts the Likert 5-point scoring method, the total score ranges from 32 to 160. A representative item was, “In online synchronous learning, the teacher organizes course content logically, with appropriate distribution of course load and knowledge points.” In the original compilation study by Cheng et al. (27), exploratory factor analysis was conducted on the scale. The results showed that the KMO value was 0.945 (greater than 0.8), and the approximate chi-square value of the Bartlett’s sphericity test was 5359.626 (with degrees of freedom of 496, *p* < 0.001), indicating that the scale has good structural validity. In this study, the Cronbach’s *α* coefficient of the scale was 0.954, this indicates that its reliability is acceptable.

#### 2.2.2 Self-regulated learning scale (SRL)

This study employed the chinese version of the online SRL questionnaire, developed by Barnard et al. (28) and translated by Zhang et al. (29). The questionnaire consists of six dimensions: goal setting (4 items, score range 4 ~ 20), environment construction (3 items, score range 3 ~ 15), task strategy (4 items, score range 4 ~ 20), time management (3 items, score range 3 ~ 15), seeking help and self-assessment(4 items, score range 4 ~ 20), self-assessment(4 items, score range 4 ~ 20)with a total of 22 items. The Likert 5-level scoring method is adopted, the total score ranges from 22 to 110. The higher the total score, the stronger the self-regulation ability of online learning. In the study by Zhang et al., this scale demonstrated good structural validity (KMO value was 0.941, greater than 0.8), and the approximate chi-square value of Bartlett’s sphericity test was 3175.632 (degrees of freedom 276, *p* < 0.001). In this study, the Cronbach’s *α* coefficient of this questionnaire was 0.922. This indicates that its reliability is acceptable. This questionnaire has been widely applied in the assessment of SRL ability of online learners in multiple studies (30).

#### 2.2.3 Academic self-efficacy scale (ASE)

The tool used in this study to assess the academic self-efficacy of nursing students is the “academic self-efficacy scale (ASE).” This scale does not directly adopt a single ready-made tool. Instead, it was compiled by Zhou et al. (31) based on the integration of multiple mature scales. Specifically, Zhou et al. (31) built upon the “Academic Self-Efficacy Scale” developed by Zhang Jianwei and Bian Yufang, and referred to the sections on learning self-efficacy in the “Motivated Strategies for Learning Questionnaire” (MSLQ) compiled by Lee et al. (32). Through project screening and revision, they ultimately formed a “Academic Self-Efficacy Scale” consisting of 7 items. This scale includes two dimensions: general self-efficacy (students’ confidence and sense of achievement in the learning process, 4 items, with a score range of 4 ~ 20) and special self-efficacy (students’ perception of their academic self-efficacy during online learning, 3 items, with a score range of 3 ~ 15). The scale uses the 5-point Likert scoring method, with values ranging from 1 (completely inconsistent) to 5 (completely consistent), the total score ranges from 7 to 35 points. A representative item was, “I think I have a strong ability to study courses independently.” The higher the score, the higher the academic self-efficacy level of nursing students. In Zhang et al.’s research, exploratory factor analysis was conducted on the scale, and two factors with eigenvalues greater than 1 were extracted, with a cumulative variance explanation rate of 68.54%. Confirmatory factor analysis showed that the two-factor model fit well (CFI = 0.975, RMSEA = 0.071, NFI = 0.963). The Cronbach’s *α* coefficient of the total scale was 0.829, and the α coefficients of each dimension were 0.833 and 0.792 respectively, indicating that the scale has good reliability and validity. In this study, the overall Cronbach’s coefficient of this scale was 0.958, which indicates that its reliability is acceptable.

### 2.3 Statistical methods

This study utilized the SPSSAU statistical analysis platform for data processing. In the model, ORTTI serves as the independent variable (X), SRL ability as the dependent variable (Y), and ASE as the mediating variable (M). The specific analysis steps were as follows: (1) The Harman single-factor test was applied to examine common method bias; (2) Descriptive statistical analysis was conducted, with categorical variables represented by frequency and percentage, and continuous variables meeting normal distribution described by mean ± standard deviation. When the average values of the total scores are both higher than half of the total score of the scale, it indicates that the overall level of nursing students in these variables is in the upper-middle range; (3) Pearson correlation analysis was used to explore the correlation among ORTTI, ASE and SRL ability; (4) 5,000 repeated samples were conducted using the Bootstrap sampling method to test the significance of the mediating effect of ASE and to calculate the 95% confidence interval. This study builds an assumption model based on established theoretical frameworks (such as social cognitive theory, self-regulated learning theory), and there is sufficient theoretical support for the relationship between the independent variable, dependent variable, and mediating variable. The demographic variables were not explicitly identified as key variables influencing these core relationships in the literature. Therefore, these demographic variables were not included in the regression or mediation models as control variables.

## 3 Results

### 3.1 General demographic characteristics of nursing students

As shown in Supplementary Table S1, a total of 813 nursing students were included in this study, among whom 128 were male (15.74%) and 685 were female (84.26%). The distribution by grade was as follows: 85 first-year students (10.46%), 348 second-year students (42.80%), 325 third-year students (39.98%), and 55 fourth-year students (6.77%). There were 319 people (39.29%) with clinical internship experience and 494 people (60.71%) without clinical internship experience. There were 378 people (46.49%) who had won awards in subject competitions and 435 people (53.51%) who had no such experience. There are 350 students (43.05%) planning to take the postgraduate entrance examination and 463 students (56.95%) planning to directly enter the workforce.

### 3.2 Common method bias test

This study employed the Harman single-factor test method to conduct exploratory factor analysis. After factor rotation processing, a total of 9 common factors with eigenvalues greater than 1 were selected. The variance contribution rate of the first factor was 39.748%, which did not exceed the critical threshold of 40%. Thus, serious common method bias was not indicated by Harman’s single-factor test (33).

### 3.3 Assessment of statistical assumptions

Before conducting the primary analyses, we assessed the normality of the data to ensure the appropriateness of using parametric tests (i.e., Pearson correlation, linear regression). The skewness and kurtosis values for all core variables are presented in Table 1. The results indicated that the absolute values of skewness for all variables ranged from 1.413 to 1.540, and the absolute values of kurtosis ranged from 0.344 to 1.049. According to established guidelines (34), skewness values within ±3 and kurtosis values within ±8 indicate acceptable univariate normality. Although the Kolmogorov–Smirnov and Shapiro–Wilk tests yielded significant *p*-values (*p* < 0.001), this is a common phenomenon in large-sample studies (N = 813) due to the high sensitivity of these tests. Given the acceptable skewness and kurtosis, along with the large sample size satisfying the central limit theorem, the data met the assumptions for Pearson correlation and multiple linear regression.

**Table 1:** Descriptive statistics and normality test results of online real-time teaching interaction, academic self-efficacy, and self-regulated learning ability among nursing students.

Variable	N	Mean	SD	Skewness	Kurtosis
Online real-time teaching interaction (ORTTI)	813	121.920	22.498	−1.540	1.049
Academic self-efficacy (ASE)	813	26.333	5.747	−1.413	0.697
self-regulated learning ability (SRL)	813	79.222	16.122	−1.435	0.344

N, sample size; SD, standard deviation. The absolute values of skewness (< 3) and kurtosis (< 8) indicate acceptable univariate normality for parametric tests.

### 3.4 Online real-time teaching interaction (ORTTI), academic self-efficacy (ASE) and self-regulated learning (SRL) ability scores among nursing students

The survey results of 813 nursing students show (see Table 2), ORTTI (3.81 ± 0.703), SRL ability (3.601 ± 0.733), and ASE (3.762 ± 0.821).

**Table 2:** Scores of online real-time teaching interaction, academic self-efficacy and self-regulated learning ability among nursing students.

Dimensionality	Score	Item mean score
Online real-time teaching interaction (ORTTI)	121.920 ± 22.498	3.810 ± 0.703
Human-media interaction	19.323 ± 3.905	3.865 ± 0.781
Teacher-student interaction	34.888 ± 6.747	3.876 ± 0.750
Interaction among students	35.096 ± 6.661	3.900 ± 0.740
Self-interaction	32.613 ± 9.140	3.624 ± 1.016
Self-regulated learning ability (SRL)	79.222 ± 16.122	3.601 ± 0.733
Goal Setting	14.643 ± 3.800	3.661 ± 0.950
Environmental structuring	10.797 ± 2.960	3.599 ± 0.987
Time management	10.622 ± 2.791	3.541 ± 0.930
Task strategies	14.526 ± 3.679	3.631 ± 0.920
Help seeking	14.300 ± 3.596	3.575 ± 0.899
Self-evaluation	14.335 ± 3.877	3.584 ± 0.969
Academic self-efficacy (ASE)	26.333 ± 5.747	3.762 ± 0.821

### 3.5 Analyze the correlation among online real-time teaching interaction (ORTTI), self-regulated learning (SRL) ability, and academic self-efficacy (ASE) of nursing students

As shown in Table 3, the SRL ability, ORTTI, and ASE of nursing students all show a significant positive correlation. Among them, the correlation coefficient between SRL ability and ORTTI was 0.809 (*p* < 0.01), and the correlation coefficient with ASE was 0.852 (*p* < 0.01).

**Table 3:** Correlation analysis of self-regulated learning ability, online real-time teaching interaction and academic self-efficacy of nursing students.

Dimensionality	Self-regulated learning ability (SRL)	Goal setting	Environmental structuring	Time management	Task strategies	Help seeking	Self-evaluation
Academic self-efficacy (ASE)	0.852**	0.689**	0.644**	0.633**	0.673**	0.705**	0.627**
Online real-time teaching interaction (ORTTI)	0.809**	0.658**	0.609**	0.610**	0.651**	0.672**	0.605**
Human-media interaction	0.721**	0.540**	0.563**	0.536**	0.592**	0.590**	0.546**
Teacher-student interaction	0.783**	0.605**	0.594**	0.582**	0.645**	0.645**	0.580**
Interaction among students	0.740**	0.568**	0.552**	0.559**	0.592**	0.615**	0.564**
Self-interaction	0.566**	0.453**	0.418**	0.434**	0.442**	0.477**	0.416**

**p < 0.01.

### 3.6 Multivariate linear regression analysis of the self-regulated learning (SRL) ability of nursing students

A multiple linear regression analysis was conducted with ORTTI and ASE as independent variables and SRL ability as the dependent variable. Prior to interpreting the regression coefficients, multicollinearity diagnostics were conducted to ensure the reliability of the model. The results showed that the Variance Inflation Factor (VIF) values for both ORTTI and ASE were 2.845, and the Tolerance values were 0.352. Since all VIF values were well below the conservative threshold of 5 and Tolerance values were greater than 0.1, severe multicollinearity was not present, indicating that ORTTI and ASE can be reliably included together as independent is associated with the model. The results show that online real-time teaching interaction and academic self-efficacy can jointly explain 76.8% of the total variation in the self-regulated learning ability of nursing students (see Table 4).

**Table 4:** Linear regression analysis results.

Predictors	*β*	*b*	*SE*	*t*	95%CI	VIF	Tolerance
Constant	-	6.525	1.503	4.340**	3.574 ~ 9.476	-	-
Online real-time teaching interaction (ORTTI)	0.351	0.251	0.020	12.307**	0.211 ~ 0.291	2.845	0.352
Academic self-efficacy (ASE)	0.570	1.598	0.080	20.000*	1.441 ~ 1.755	2.845	0.352
∆*R*^2^	0.768						
F	*F* (2,810) = 1348.309 *p* = 0.000						

*p < 0.05; **p < 0.01 Durbin-Watson = 2.026. B, unstandardized coefficient; SE, standard error; β, standardized coefficient; CI, confidence interval; VIF, variance inflation factor. Tolerance > 0.1 and VIF < 5 indicate no severe multicollinearity.

### 3.7 Analysis of the mediating effect of academic self-efficacy (ASE) between online real-time teaching interaction (ORTTI) and the self-regulated learning (SRL) ability of nursing students

**Figure 1:**
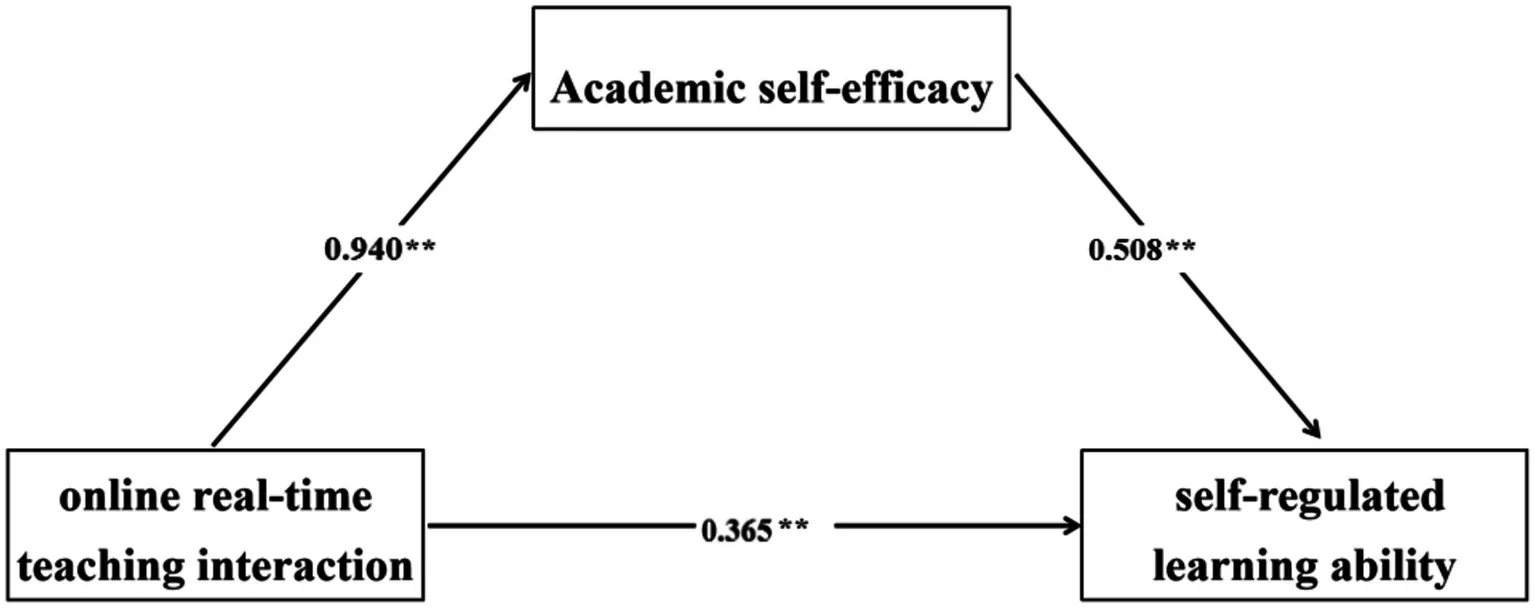
Path diagram of the mediating effect of academic self-efficacy (ASE) between online real-time teaching interaction (ORTTI) and self-regulated learning ability (SRL). All path coefficients represent standardized estimates derived from the Bootstrap mediation analysis. **p < 0*.001*.

To examine the impact of ORTTI on the SRL ability of nursing students, a mediation analysis based on the Bootstrap method was conducted. In this model, ORTTI was set as the independent variable, ASE was set as the mediating variable, and SRL ability was set as the dependent variable (Figure 1). The results showed that the mediating effect value of ASE between ORTTI and SRL ability was 0.478 (*p* < 0.01), and its 95% confidence interval was [0.385, 0.520], excluding 0, indicating that this mediating effect reached a significant level. Meanwhile, the direct effect of ORTTI on SRL ability was 0.365 (p < 0.01). The proportion of indirect effects in the total effect is 56.7% (Table 5). In conclusion, ASE plays a key mediating role between ORTTI and SRL ability.

**Table 5:** Results of bootstrap mediation effect test.

Parameters	Estimates of value	Lower limit	Upper limit	*p*	Effect proportion/%
Indirect effect	0.478	0.385	0.520	<0.001	56.7
Direct effect	0.365	0.307	0.424	<0.001	43.3
Total effect	0.843	0.801	0.886	<0.001	100

Bootstrap sample size = 5,000. CI, confidence interval. The indirect effect is significant as the 95% CI does not include 0.

## 4 Discussion

### 4.1 The self-regulated learning (SRL) ability of nursing students

Descriptive analysis showed that the overall online SRL scores of nursing students were slightly above the theoretical median, indicating a moderate level of SRL. This is generally consistent with previous studies among online learners, including nursing and health profession students, suggesting room for further development of SRL abilities (25, 29, 35). Regarding specific SRL dimensions, scores varied across goal setting, task strategies, environment structuring, self-evaluation, help seeking, and time management. While goal setting received relatively higher scores, this should be interpreted as an indication of its potential importance for nursing students’ SRL, rather than implying a causal effect on learning outcomes. Differences across dimensions may reflect variations in professional background, learning motivation, prior experiences, and the online learning environment. For instance, nursing students’ clinical goals and professional expectations may be associated with higher engagement in goal-setting behaviors, while time management and help-seeking strategies may require additional support. These findings highlight that certain aspects of SRL, particularly goal setting, may support students’ academic self-efficacy and intrinsic motivation, contributing to more effective online learning experiences. Future studies could further explore how individual SRL dimensions relate to learning outcomes in nursing education contexts.

### 4.2 Online real-time teaching interaction (ORTTI) was positively associated with the self-regulated learning (SRL) ability of nursing students

According to social cognitive theory, SRL exhibits distinct social characteristics (36). This means that learners’ SRL behaviors are largely achieved through interactions with the external environment, encompassing processes such as self-observation, self-evaluation, and self-regulation (37). The interaction in online learning has a significant impact on SRL (38). Therefore, teaching interactions can be regarded as a key environmental variable in online SRL. In this study, the overall level of online real-time teaching interaction among college nursing students was relatively good. The total score of the scale was 121.920 ± 22.498, and the average individual score was 3.810 ± 0.703, which was at a medium to high level. Regarding the structural characteristics of interaction types, scores for teacher-student interaction (3.876 ± 0.750) and student–student interaction (3.900 ± 0.740) were particularly prominent. This indicates that within synchronous online teaching environments, these two forms of interaction remain core elements for constructing effective learning experiences. In contrast, the self-interaction dimension scored the lowest (3.624 ± 1.016). Given the pivotal role of instructional interaction in online learning, it is particularly important to foster higher levels of the level of interaction in online learning, especially by refining the design and organization of self-interaction. The correlation analysis revealed a significant positive association between ORTTI (the overall score and the four dimensions) and SRL. The higher the level of interaction, the stronger the self-regulation ability (36). From a practical perspective, this finding implies that nursing educators should consciously design interactive activities (such as real-time case discussions, role-playing scenarios, or collaborative problem-solving) to provide social scaffolds that support SRL (39).

### 4.3 The mediating role of academic self-efficacy (ASE) between online real-time teaching interaction (ORTTI) and self-regulated learning (SRL) ability among nursing students

The results indicate that academic self-efficacy exerts a significant mediating effect between online real-time teaching interactions and SRL abilities among college nursing students, highlighting its pivotal role in facilitating students’ SRL processes. This suggests that online real-time teaching interactions are positively associated with higher nursing students’ ASE, which is linked to their proactive learning behaviors, and ultimately is associated with better development of their SRL abilities. According to social cognitive theory, an individual’s evaluation of their own abilities, namely self-efficacy, as a core element in the self-regulation system, has a significant impact on the learning process (36). Self-efficacy mainly regulates learning outcomes by influencing students’ cognitive engagement and strategy usage. Students with higher self-efficacy tend to set clear learning goals, arrange learning tasks reasonably, maintain a high level of learning motivation, and adjust their learning strategies flexibly. Such students usually demonstrate stronger learning motivation and more efficient self-regulated ability (40).

This intermediary approach is particularly prominent in nursing education. Contemporary health science research has confirmed that structured online interaction and timely feedback can be positively associated with higher academic confidence of nursing students, which in turn translates into more effective self-regulated theoretical and clinical learning (12, 25). The nursing knowledge system is rigorous, has many practical difficulties, and has strong theoretical abstraction. Nursing students face unique academic pressure. In the online nursing education scenario, diverse real-time interaction can exert its efficacy by reshaping students’ learning cognition and confidence. Real-time feedback and targeted guidance from teachers, as well as peer recognition and experience support, can effectively alleviate the fear of nursing students when facing complex professional knowledge, help students accumulate phased learning success experiences, and gradually establish stable academic self-efficacy; while students with high academic self-efficacy will actively set clear learning goals, flexibly adjust learning strategies, actively engage in self-monitoring and assessment, and ultimately are associated with higher SRL capabilities (36).

Based on these findings, nursing education practitioners should fully recognize the educational value of ORTTI. They should effectively leverage its advantages such as flexibility, convenience, strong interactivity, and timely feedback. Teachers can design systematic classroom interaction activities. On the one hand, this is associated with higher enthusiasm of nursing students in participating in the class. On the other hand, it enables them to monitor the students’ learning progress in real time and dynamically optimize the teaching plan based on the actual learning situation. This approach is associated with optimized learning experiences and stronger academic self-efficacy, which in turn supports the development of their SRL abilities.

## 5 Practical implications

First, the course development team should design interactive tasks centered around real clinical problems or typical nursing cases, aligning them with higher-order thinking objectives to foster nursing students’ motivation for active inquiry. In the process of task design, it is necessary to fully consider the differences in students’ prior knowledge and cognitive levels, and set a gradient of task difficulty. This not only avoids cognitive overload caused by a single task but also prevents learning frustration due to excessively high difficulty. Second, institutions should optimize the interactive functions of online teaching platforms according to their available resources. For universities with advanced technical support, implementing features like virtual breakout rooms, real-time collaboration tools, and visual learning analytics dashboards can serve as highly effective, data-driven strategies. However, for institutions with limited resources, low-cost but impactful alternatives should be prioritized. These include structured peer discussions, short formative quizzes embedded within learning modules, and targeted teacher feedback prompts. Third, to directly address our finding that the self-interaction dimension scored the lowest (3.624 ± 1.016), nursing educators should implement specific scaffolding tools to foster students’ internal dialog and metacognitive monitoring. We recommend integrating structured self-reflection activities, guided learning logs, self-assessment checklists, and clinical goal-setting templates into the online nursing curriculum. For instance, providing a standardized ‘weekly self-reflection log’ can guide students to evaluate their learning strategies and identify areas for improvement after each online module. Finally, establish a formative assessment and dynamic optimization mechanism spanning the entire teaching process. By systematically collecting platform learning behavior data, conducting regular learning experience surveys, and integrating classroom observations and in-depth interviews from teaching supervisors, a multi-source, mutually corroborating evaluation system can be constructed. This enables multidimensional diagnosis and continuous improvement of teaching effectiveness, forming a virtuous cycle of “design-implementation-evaluation-optimization.”

These practical implications are primarily derived from the context of Chinese nursing education. While they offer valuable insights for similar educational settings, educators and policymakers in other countries or cultural contexts should adapt these strategies to align with their specific institutional resources, technological infrastructure, and pedagogical traditions.

## 6 Limitations

A major limitation of this study is its cross-sectional design. Because the data were collected at a single time point, we cannot definitively establish the temporal order or causal relationships among online real-time teaching interaction, academic self-efficacy, and SRL ability. Although our multiple linear regression and Bootstrap mediation analyses support the hypothesized mediating pathways, alternative explanations, such as reverse causality, cannot be entirely ruled out. Therefore, future longitudinal or experimental studies are strongly recommended to clarify the causal direction and the dynamic, reciprocal interplay underlying these relationships.

Due to the limitations of sampling conditions, a convenience sampling method was adopted, and the samples for this study were exclusively sourced from undergraduate nursing students in specific colleges and universities in Jilin Province. This non-random nature of the sample limits its representativeness and may introduce selection bias. Consequently, the findings may not be readily generalizable to nursing students in other regions or educational contexts. Differences in institutional resources, the quality of online teaching platforms, the level of teacher training in digital pedagogy, and students’ varying access to technology and stable internet connections may be significantly associated with the relationships observed in this study. Therefore, the extrapolation of the current research conclusions should be treated with caution. Future research should expand the sampling scope to encompass universities of varying geographic locations, types, and levels, and employ probability sampling methods wherever feasible, thereby testing the model’s universality and stability across diverse educational contexts.

Furthermore, although Harman’s single-factor test indicated no severe common method bias, this statistical procedure cannot entirely rule out the possibility that the observed correlations were partially inflated by the shared measurement method. Additionally, relying solely on scales is insufficient for capturing the dynamic psychological constructs and behavioral adjustment mechanisms occurring during teaching interactions. Future studies should consider employing multi-source data collection or a mixed-methods research approach, integrating in-depth interviews and teaching record observations, to provide richer empirical support for theoretical development.

Finally, we acknowledge the notably high correlation coefficients observed among the core variables. We have carefully examined the scales and confirmed that there is no conceptual overlap or item similarity, as they measure distinct constructs based on social cognitive theory: the interactive environment (ORTTI), motivational beliefs (ASE), and learning strategies (SRL). The strong correlations can be theoretically explained by Bandura’s triadic reciprocal determinism, where environment, personal factors, and behavior are highly synergistic in online learning contexts. Moreover, the Harman’s single-factor test (first factor = 39.748% < 40%) and multicollinearity diagnostics (VIF = 2.845 < 5) confirmed that shared method variance and severe multicollinearity did not artificially inflate these associations. The robust Bootstrap confidence intervals further support the reliability of the mediation interpretation. Nevertheless, we acknowledge that such high correlations warrant cautious interpretation, and future studies should employ more distinct measurement tools or multi-source data to further verify these relationships.

## Data Availability

The original contributions presented in the study are included in the article/Supplementary material, further inquiries can be directed to the corresponding author/s.
