# Supplementary material for: Online real-time teaching interaction and self-regulated learning ability: a cross-sectional mediation study among undergraduate nursing students
Source: Front Public Health. 2026 Sep 7;14:1872098. doi: 10.3389/fpubh.2026.1872098 (PMC13590722; doi:10.3389/fpubh.2026.1872098)
Supplement: Supplementary file 1 [file Data_Sheet_1.PDF]

Supplementary Material

1 Table S1

Characteristic	Category	n	%
**Gender**	Male	128	15.74
	Female	685	84.26
**Grade**	First-year	85	10.46
	Second-year	348	42.80
	Third-year	325	39.98
	Fourth-year	55	6.77
**Clinical Internship Experience**	Yes	319	39.29
	No	494	60.71
**Subject Competition Awards**	Yes	378	46.49
	No	435	53.51
**uture Plan**	Postgraduate entrance examination	350	43.05
	Direct employment	463	56.95
